# Traumatic injury in female *Drosophila melanogaster* affects the development and induces behavioral abnormalities in the offspring

**DOI:** 10.1186/s12993-019-0163-1

**Published:** 2019-10-25

**Authors:** Ved Chauhan, Abha Chauhan

**Affiliations:** Department of Neurochemistry, New York State Institute for Basic Research in Developmental Disabilities, 1050 Forest Hill Road, Staten Island, NY 10314 USA

**Keywords:** Developmental disorders, *Drosophila melanogaster*, Larval length, Pregnancy, Social interaction, Traumatic injury

## Abstract

Traumatic injury (TI) during pregnancy increases the risk for developing neurological disorders in the infants. These disorders are a major concern for the well-being of children born after TI during pregnancy. TI during pregnancy may result in preterm labor and delivery, abruptio placentae, and/or fetomaternal hemorrhage. *Drosophila melanogaster* (fruit fly) is a widely used model to study brain and behavioral disorders in humans. In this study, we analyzed the effects of TI to female fruit flies on the development timing of larvae, social interaction and the behavior of offspring flies. TI to the female flies was found to affect the development of larvae and the behavior of offspring flies. There was a significant increase in the length of larvae delivered by traumatically injured maternal flies as compared to larvae from control maternal flies (without TI). The pupae formation from larvae, and the metamorphosis of pupae to the first generation of flies were faster in the TI group than the control group. Negative geotaxis and distance of the fly to its nearest neighbor are parameters of behavioral assessment in fruit flies. Negative geotaxis significantly decreased in the first generation of both male (p = 0.0021) and female (p = 0.0426) flies. The distance between the first generation of flies to its nearest neighbor was shorter in both male and female offspring flies in the TI group as compared to control group flies. These results indicate that TI to the female flies affected the development of larvae and resulted in early delivery, impaired social interaction and behavioral alterations in the offspring.

## Background

The Centers for Disease Control and Prevention (CDC) estimates the prevalence of developmental disorders at 1 in every 6 children [1]. Neurodevelopmental disorders such as intellectual disability, attention-deficit hyperactivity disorder, autism spectrum disorder (ASD), and fragile X syndrome have core abnormal behavioral components that are fundamental to their diagnosis. Abnormal social interactions and impairments in verbal and non-verbal communication as well as repetitive and restricted behavior or interests are core components in autism diagnosis. In addition, other features are often associated with this triad, such as difficulties with decision-making [2].

Traumatic injury (TI) during pregnancy is a risk factor for neurological disorders in children. The injuries during pregnancy occur by motor vehicle accidents (49%), falls (25%), assaults (18%), gun shots (4%), and burns (4%) [3]. Up to 6–7% of pregnancies are affected by some degree of TI [4], which is usually accidental but may include intentional violence. TI during pregnancy may lead to preterm labor and delivery, abruptio placentae, and/or fetomaternal hemorrhage [5–9]. TI in pregnant women increases the risk for several mental and physical disabilities, which include developmental delay, vision and hearing loss, speech impairment, learning and communication difficulties, movement abnormalities, and mental health issues in the children [10–13].

*Drosophila melanogaster* is a widely used model for studies of brain disorders such as Parkinson’s disease, Alzheimer’s disease, Huntington’s disease, fragile X syndrome, and Angelman syndrome [14]. It is a comparatively simple organism, with its genome consisting of four chromosomes encoding approximately 14,000 genes [15, 16]. On the other hand, there are 20,000 protein and RNA- coding genes in the human genome [17]. Among 59 of the human neurological genes examined, 38 have orthologs in the *Drosophila* genome [18]. A single *Drosophila* gene may serve the same function as multiple related genes of mammals, thus decreasing the redundancy seen in other vertebrate models. Although flies and humans are distinctly different from each other, many molecular processes are conserved between them. The advantage of studying neurobehavioral disorders in *Drosophila* is the presence of genes that are similar to human genes for normal cognitive functions [19, 20]. The *Drosophila* model exhibits complex behaviors relevant to humans, including courtship [21, 22], circadian rhythms [23], learning and memory [24], aggression [25], grooming [26], and open field exploration [27]. It is also an attractive model because of its quicker generation time, large number of progeny for better selection, and easy maintenance.

There are differences between pregnant fruit flies and pregnant women. Fruit flies deliver their embryos, after which the development of embryo to fruit fly takes place outside, whereas embryo-to-infant development takes place inside the uterus in women. Therefore, the fruit fly is considered a good model to study the transition between stages, i.e., embryo; 1st, 2nd, and 3rd instar larva; and pupa to fly. We have previously reported that environmental risk factors cause neurobehavioral changes in *Drosophila*. In fruit flies, we observed that exposure to bisphenol A (BPA) affects behavior [28], and that methylmercury exposure inhibits alcohol dehydrogenase [29] and affects sexual functions [30].

In this study, we have examined the effects of TI to female flies on the development of larvae and the behavior of the first generation of flies. Climbing or negative geotaxis is an innate behavior of the fruit fly. There has been considerable interest in using this simple behavior to gain insight into the alterations in brain function [31]. Therefore, we compared negative geotaxis in TI and control offspring flies to study whether TI to female flies induces behavior abnormalities in the offspring. We also studied the effects of TI on social interaction among the offspring flies by measuring the distance between a fly and its nearest neighbor in a social space assay, as described previously [28, 32].

## Materials and methods

### *Drosophila melanogaster*

Wild-type Oregon-R *Drosophila melanogaster* stocks were maintained at 25^0^ C on a standard cornmeal diet (Jazz-mix *Drosophila* food, Fisher Scientific, Pittsburgh, PA, USA) under 12 h:12 h light and dark cycle.

### Traumatic injury to female flies

**Fig. 1:**
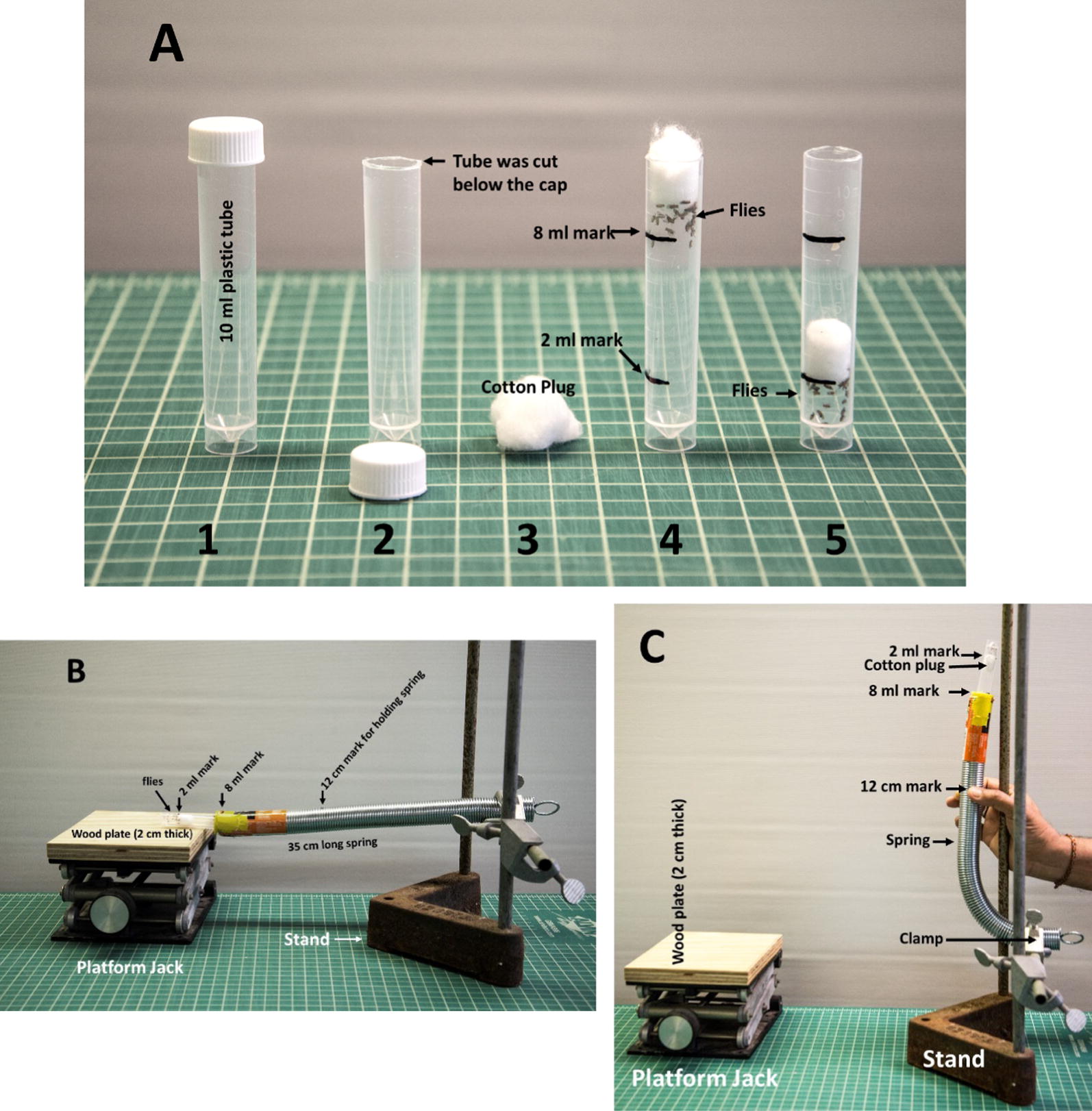
Procedure of inducing traumatic injury in flies. A plastic tube (10 ml) was cut below the cap, and it was marked at 2 ml and 8 ml. The flies were transferred into the tube and pushed down to the 2-ml mark by a cotton plug (**A**). The plastic tube was then inserted into a spring up to the 8-ml mark (**B**). As shown **C**, the spring was then lifted by holding the spring at its 12-cm mark so that the spring could touch the stand. After that, the spring was released to hit the platform jack, which had a 2-cm-thick wooden plate

As shown in Fig. 1, 10-ml polypropylene tubes (Genesee Scientific; catalogue number 21-392) (Fig. 1A, #1) were cut below the cap (Fig. 1A, #2), and a cotton plug (Fig. 1A, #3) was placed. The tube was marked at 2 ml and 8 ml (Fig. 1A, #4) Twenty female flies (6–8 days old) were separated from male flies just before they were transferred to these tubes. The flies were brought down to the 2-ml mark on the tubes by tapping the tube and pushing down the cotton plug (Fig. 1A, #5). We induced TI to female flies following the method of Katenberger [33]. As described in Fig. 1B, C, the top end of the tube was slid in the spring to the 8-ml mark, and then the spring was released to hit the platform jack to induce TI to the flies.

The female flies were transferred to food vials at 7 pm immediately after TI. At the same time, 20 control flies (without TI) of the same age were also transferred to food vials. The next day at 7 am, the flies were removed, and the development of larvae was studied.

In this report, larvae, pupae and offspring flies that emerged from maternal flies after TI are identified as TI-larvae, TI-pupae and TI flies. Similarly, larvae, pupae and flies obtained from control female flies are identified as C-larvae, C-pupae and C-flies.

### Handling of larvae

When larvae were 4 days old (3rd instar larvae), 20% sucrose solution was added to the food vials, and the floating larvae were removed after 5 min. The larvae were placed on glass slides and kept in a refrigerator. Photographs of the larvae along with a scale were taken.

### Measurement of length of larvae

The changes in larval length in Drosophila may indicate the changes in development. We measured the length of larvae using ImageJ NIH software (http://imagej.nih.gov/ij/). In brief, a file was opened in ImageJ. A straight line was selected, and a line (1-cm- long) on the scale was drawn, followed by clicking ‘analyze’ and ‘measure’. Next, ‘set scale’ was clicked; followed by clicking ‘change distance’ to 1 cm (do not change distance in pixels, and ‘aspect ratio’), and then ‘OK’ was clicked. Then ‘analyze’ followed by ‘measure’ was clicked. This technique produces a 1-cm-length measuring tool. Afterwards, a line (straight or free-hand line) was drawn on the larval length, and ‘measure’ was clicked each time, yielding the length of larvae in cm.

### Time taken for origination of flies from pupae

The control and TI flies were placed in the food vials to lay eggs for 12 h from 7 pm to 7 am the next day. The flies were removed at 7 am, and pupae-to-fly conversion, i.e., eclosion of flies was counted each morning.

### Negative geotaxis

In one set of experiment, 20 flies of the first generation (6–8 days old) were transferred to a *Drosophila* vial (narrow), and another vial was placed over it. These two vials were then joined with tape. To count the number of flies crossing 3/4th of the distance in the two vials, a line was drawn at the ¾^th^ distance (14.5 cm) on the two vials. The tubes were tapped five times, and movement of the flies was video-recorded. The number of flies crossing the 14.5-cm mark was counted. One more set of experiments with different group of flies was conducted in a similar manner.

### Social interaction

Social interaction was determined in male and female flies by using the social space assay, as we described previously [28]. The plastic chamber used for this assay had a triangle of 16 cm base and 17 cm height, which was covered from both sides by square plastic plates (18 cm × 18 cm). In brief, the first generation of flies (6–8 days old) from the control (n = 20) and TI groups (n = 20) were separated according to their gender, and transferred to the chamber. After the flies spent 40 min exploring and settling in the chamber, a digital image of the chamber with the flies was taken with a camera. Image J was then used to process the image into an 8-bit binary image. The binary image was then imported to the Lispix program (NIH image analysis software—http://www.nist.gov/lispix/) to calculate the distance of the fly to its nearest neighboring fly.

### Data analysis

Data were analyzed for larval size by Student *t* test, and for negative geotaxis and social interaction (distance of the fly to its nearest neighbor fly) by one-way ANOVA using GraphPad Prism 10.

## Results

### Effect of traumatic injury on survival rate of female flies

It was observed that 20–30% of traumatically injured female flies died. Therefore, the number of maternal flies after TI was always less than the number of the control maternal flies. However, we used the same number of control and TI offspring flies in each assay.

### Traumatic injury affects larval length

**Fig. 2:**
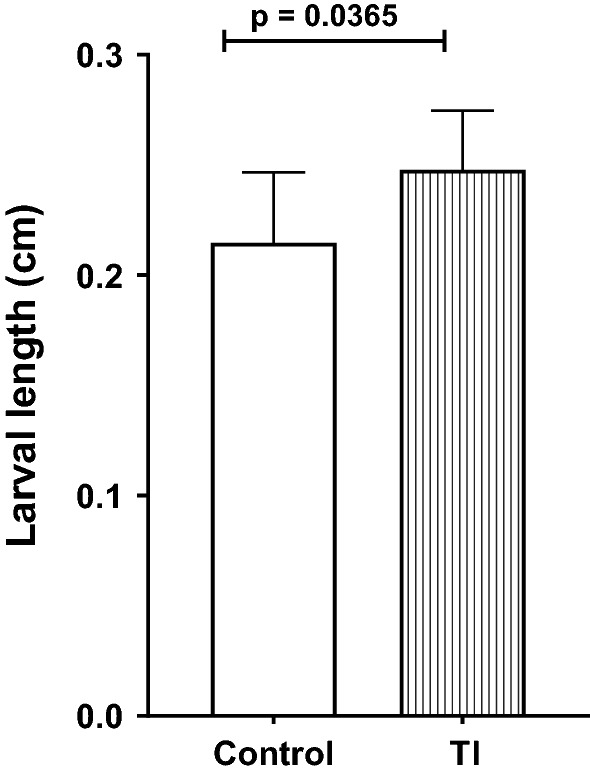
Effect of traumatic injury to female flies on the length of larvae. The length of larvae in the TI and control groups was measured. Twenty larvae in each group were used to measure the length of larvae. There was a significant increase in the larvae length of TI-larvae (larvae obtained from traumatically injured female flies) as compared to control larvae (larvae obtained from female flies without TI). The data were analyzed with Student t-test

The larvae (4 days old) were collected and their length was measured as described in Methods section. TI to female flies resulted in a significant increase (p = 0.0365) in larval length (0.2478 ± 0.026 cm, mean ± SD, N = 20) as compared to that in control flies (0.2147 ± 0.31 cm, mean ± SD, N = 20) (Fig. 2). This data suggests that the development of the larvae gets accelerated in the TI group.

### *Traumatic injury in female flies causes early metamorphosis from pupa to* fly

**Fig. 3:**
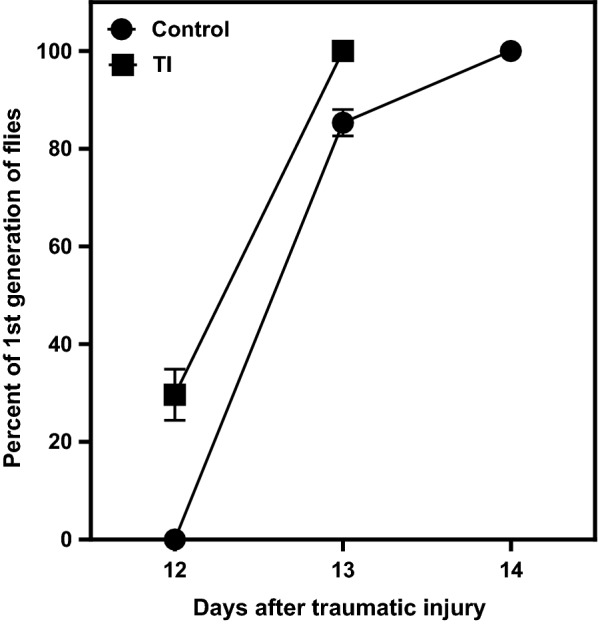
Origination of flies from traumatic injury-pupae (pupae obtained from traumatically injured female flies) and control- pupae (pupae obtained from female flies without TI). TI caused a shift in the timing of metamorphosis. In the TI group, flies started emerging 1 day earlier than flies in the control group. Three sets of independent experiments were done in each group

Metamorphosis of flies, i.e., pupa to fly conversion was studied in TI and control flies. In the TI female flies group, pupae formation was earlier by a day and the metamorphosis started on the 12th day after TI to female flies (30% of the first generation of flies were observed) and 100% of flies were seen by the 13th day. On the other hand, no first generation of flies were seen on the 12th day in the control female flies group. Metamorphosis in control flies was observed on the 13th and 14th days. Our results showed that TI to the female flies causes a shift in the timing of metamorphosis (Fig. 3). We also observed that total number of offspring flies in TI group were slightly less as compared to control group.

### Effect of traumatic injury on negative geotaxis in first generation of flies

**Fig. 4:**
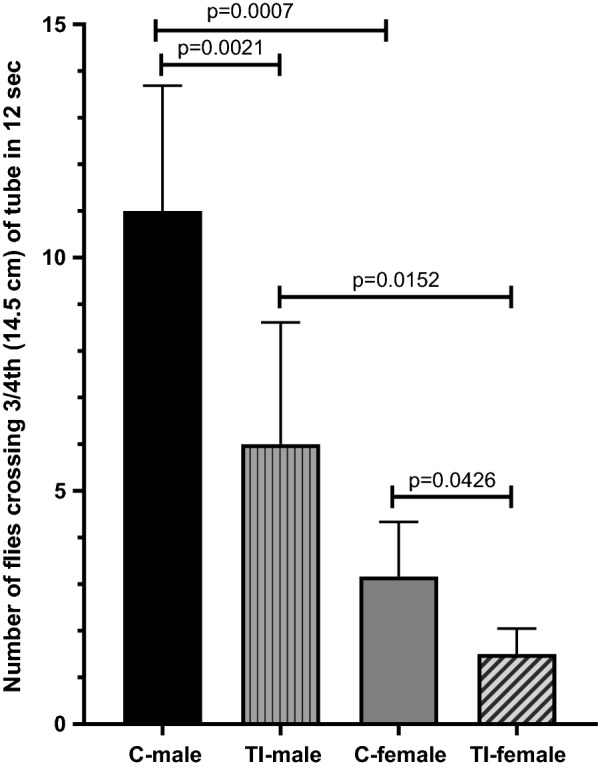
Effect of TI to female flies on negative geotaxis in first generation of flies. Negative geotaxis was significantly reduced in both male and female flies that were born after TI to female flies. C, control; TI, traumatic injury. The data were analyzed with one-way ANOVA by using the Sidak multiple comparison test. Two independent sets of experiments were done

The movement of flies in an upward direction (climbing) is called negative geotaxis. The negative geotaxis assay is important for studying behavioral changes in fruit flies. TI in female flies affected the negative geotaxis in the first generation of both male and female flies (Fig. 4). A significant reduction in negative geotaxis was observed in both TI male (p = 0.0021) and TI female flies (p = 0.0426) compared to their respective control groups. Twenty male flies and 20 female flies in each control and TI group were studied. In male flies, 6.14 ± 2.4 TI flies (mean ± SD) crossed the ¾th mark as compared to 11.4 ± 2.7 (mean ± SD) flies in the control group. In female flies, 1.57 ± 0.78 TI flies (mean ± SD) crossed the ¾th mark as compared to 3.28 ± 1.1 (mean ± SD) flies in the control group. It was also observed that negative geotaxis was significantly greater in male flies than in female flies in both the TI (p = 0.0152) and the control group (p = 0.0007).

### Abnormal social Interactions in first generation of male and female flies after traumatic injury to female flies

**Fig. 5:**
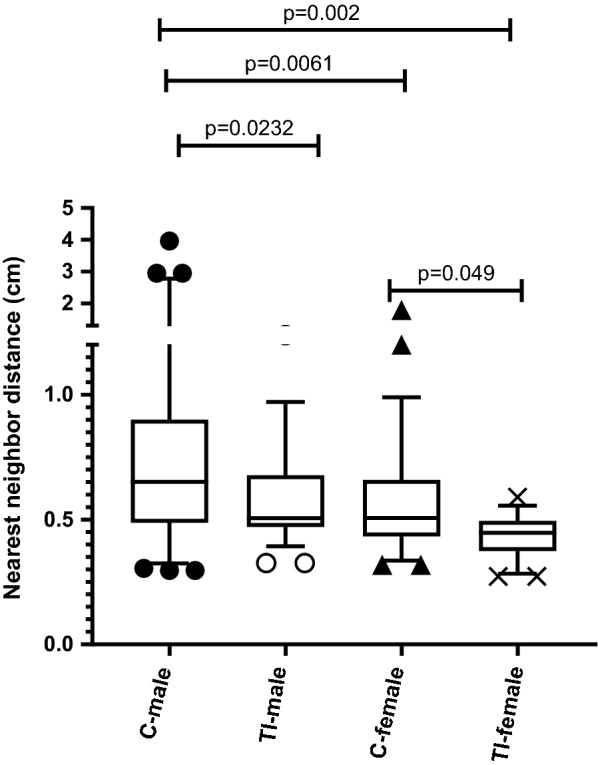
TI decreases the distance between a *Drosophila* fly and its closest neighbor in a social setting. The data are shown as box and whisker plots of the distance to the closest neighbor in the chamber, with the box representing the first quartile (25th percent) and the 3rd quartile (75th percent), the line in the box representing the median, and the Tukey whiskers excluding the outliers. These data were obtained from two independent repeats of 20 flies per assay. Data were analyzed by one-way ANOVA using the Sidak multiple comparison test

The distance between an individual fly and its closest neighbor has been used as a measure of social interaction within the group, as previously described [30, 34]. TI in female flies caused a significant effect on the distance maintained between the male and female flies (first generation) in a social group (Fig. 5). The first generation of male and female flies that originated from traumatically injured female flies was significantly closer in distance than in control flies (male flies, p = 0.0232; female flies, p = 0.049). Two sets of independent experiments with different flies were conducted in each group.

## Discussion

It has been suggested that trauma complicates approximately 1 in 12 pregnancies [34], and it is the leading non-obstetric cause of maternal death [35]. Trauma also has fetal implications and has been reported to increase the incidence of spontaneous abortion, preterm premature rupture of membranes, preterm birth, uterine rupture, cesarean delivery, placental abruption and stillbirth [36–40]. The rate of fetal death from maternal trauma is 2.3 per 100,000 live births [39], with placental abruption as a major contributing factor [41]. It is reported that 1 in 3 pregnant women admitted for trauma to the hospital delivers during her hospitalization [42]. While pregnancy per se does not appear to increase morbidity or mortality due to trauma, the presence of a gravid uterus alters the pattern of injury [41].

*Drosophila melanogaster* has been used as an animal model for traumatic brain injury [33]. In this study, we used this model to study how TI in pregnant female flies affects the offspring. We had anticipated that TI to female flies would lead to a decrease in larval length. However, to our surprise, we observed an increase in TI-larval length. TI-larvae also had faster pupae formation and metamorphosis (pupa to fly conversion) as compared to control larvae. Because pupae formation was faster in TI-larvae, we also observed a shift in metamorphosis, i.e., early metamorphosis by at least 1 day in TI-pupae than in the C-pupae. The reason for this rapid growth may be that TI in maternal flies led to faster delivery of embryos or that inflammation was increased as compared to control flies.

We also studied whether the behavior of offspring flies born after TI to female flies would be affected. Our results indicate that TI to female flies resulted in decreased negative geotaxis in both male and female flies, suggesting that TI in pregnant flies may also affect the behavior of the offspring. Not only was the negative geotaxis decreased but the distance between the nearest neighbors in flies was also decreased in the first generation of both male and female flies after TI to female flies as compared to control group without TI, suggesting that TI in flies leads to impaired social interaction in the offspring. We are not yet certain whether negative geotaxis has any direct link to social interaction, i.e., altered nearest neighbor distance of the flies. Since both these parameters are on behavioral changes in fruit fly, there is a possibility that alterations in these assays (negative geotaxis and social space assay) caused by TI to female flies may have some association.

One of the core features of autism and other neurodevelopmental disorders such as fragile X and Angelman’s syndromes is the inability of the individual to interact socially with other individuals [43–45], which is a diagnostic criterion in autism. It is also a common feature in studies using mouse models for autism [46]. Social behavior is the ability of conspecifics to interact, leading to changes in the subsequent behaviors of the individual [47–49]. In a social setting, the individual maintains a personal space or distance from another individual (personal space boundary), but also a spatial proximity to another individual for effective communication [50]. This social space or the space between two individuals of the same species is seen in most animals such as birds, fish, or locusts. When placed in a social setting, flies tend to arrange themselves uniformly rather than in aggregates or randomly [51, 52], and this social interaction in the group leads to learning of higher behaviors from their conspecifics [47, 52, 53]. Studies have shown that social isolation in *Drosophila* reduces the fiber number in the mushroom bodies, the functional equivalent of the hippocampus [54]; increases aggression [55–57]; and shortens the life span [57]. When placed in settings where they can freely interact with other flies, *Drosophila* flies usually maintain a distance of about two-body lengths (1–5 mm) among themselves, similar to other animals [32, 52]. This allows for the flies to orient themselves to interact with each other. Thus, interaction between flies follows a non-random pattern [58]. In our study, inter-fly distance was used as a measure for social interaction [59–62]. We have previously reported a decrease in the inter-fly distance when the flies exposed to BPA were placed in a social setting [28]. This decrease may be due to aberrant social interaction, in which the flies do not maintain the ideal balance of attraction/repulsion and interact inappropriately with each other. It is quite possible that children born after TI may also exhibit lack of social interaction features as observed in autism and fragile X syndrome.

## Conclusion

In female fruit flies, TI affected the larval length and metamorphosis (pupa to fly conversion), thus affecting the development of offspring. In addition, the behavior of flies born after TI to maternal female flies was also affected as assessed by negative geotaxis and social interaction (distance between the fly and its nearest neighbor) assays. These results suggest that TI in female flies causes developmental abnormalities in offspring, which results in abnormal behavioral functions of adult flies. Therefore, TI during pregnancy in human may play a major role in affecting the development and behavior of children.

### Data analysis

Data were analyzed for larval size by Student t-test, and for negative geotaxis and social interaction (distance of the fly to its nearest neighbor fly) by one-way ANOVA using GraphPad Prism 10. All the comparisons were tested and only significant comparisons are labelled.

## Data Availability

The datasets used and/or analyzed during the current study are available from the corresponding author upon reasonable request.

## Abbreviations

ASD: autism spectrum disorder
BPA: Bisphenol A
CDC: Centers for Disease Control and Prevention
TI: traumatic injury
